# Supplementary material for: Causal role of metabolites in non-small cell lung cancer: Mendelian randomization (MR) study
Source: PLoS One. 2024 Mar 22;19(3):e0300904. doi: 10.1371/journal.pone.0300904 (PMC10959361; doi:10.1371/journal.pone.0300904)
Supplement: Attachment — Submitted filename: Response to Reviewers.docx [file pone.0300904.s005.docx]

**Dear Editor**,

We appreciate you and the reviewers for the constructive comments on our manuscript titled "Causal role of metabolites in Non-small cell lung cancer: Mendelian randomization (MR) study" . Those suggestions are all valuable and very helpful for revising and improving our paper. We have studied the comments carefully and have made revision accordingly. In the revised manuscript, we have responded to the reviewers' comments point-by-point.

**The following content has been revised according to the suggestions of this reviewer, point by point.**

**Reviewer #1**:

1. Perform a reverse MR analysis to test the reverse direction relationships (NSCLC risk on metabolites) to further validate directionality

Reply: This article has already conducted a reverse Mendelian randomization, with Fig. 5 visualizing the results. The reverse Mendelian randomization suggests that there is no causal relationship between them.

2. Discuss implications of ethnicity limitations as European population genetics were used and may not extrapolate to all ancestries

Reply:The impact of racial limitations has already been addressed in the last paragraph of the discussion section of this article.

3. Comment on how conservative multiple testing correction thresholds, while rigorous, may increase false negative rates missing some true causal signals

Reply:Cite recent similar MR studies on metabolite relationships to better differentiate the novel contributions made

4.Cite recent similar MR studies on metabolite relationships to better differentiate the novel contributions made

Reply:After reviewing literature from the past five years on PubMed, we are currently the first to study Mendelian randomization of 1400 metabolites.

We appreciate the reviewers' close examination of our manuscript. We have tried our best to improve the presentation and quality of this paper according to the constructive suggestions. The revised manuscript will definitely be improved by addressing these concerns. We hope that the revision is now acceptable for publication in PLOS ONE. If you have any other concerns, please let us know. We look forward to hearing from you soon. Sincerely,

[QianLi]

On behalf of all authors
